# Resuscitating Cardiopulmonary Resuscitation Training in a Virtual Reality: Prospective Interventional Study

**DOI:** 10.2196/22920

**Published:** 2021-07-29

**Authors:** Janaya Elizabeth Perron, Michael Jonathon Coffey, Andrew Lovell-Simons, Luis Dominguez, Mark E King, Chee Y Ooi

**Affiliations:** 1 Discipline of Paediatrics School of Women's and Children's Health University of New South Wales Randwick Australia; 2 Medicine Education Development Unit Faculty of Medicine University of New South Wales Sydney Australia; 3 Educational Delivery Services Office of the Pro Vice-Chancellor (Education) University of New South Wales Sydney Australia

**Keywords:** pediatrics, cardiopulmonary resuscitation, virtual reality, medical education

## Abstract

**Background:**

Simulation-based technologies are emerging to enhance medical education in the digital era. However, there is limited data for the use of virtual reality simulation in pediatric medical education. We developed Virtual Doc as a highly immersive virtual reality simulation to teach pediatric cardiopulmonary resuscitation skills to medical students.

**Objective:**

The primary objectives of this study were to evaluate participant satisfaction and perceived educational efficacy of Virtual Doc. The secondary aim of this study was to assess the game play features of Virtual Doc.

**Methods:**

We conducted a prospective closed beta-testing study at the University of New South Wales (Sydney, Australia) in 2018. All medical students from the 6-year undergraduate program were eligible to participate and were recruited through voluntary convenience sampling. Participants attended a 1-hour testing session and attempted at least one full resuscitation case using the virtual reality simulator. Following this, participants were asked to complete an anonymous postsession questionnaire. Responses were analyzed using descriptive statistics.

**Results:**

A total of 26 participants were recruited, consented to participate in this study, and attended a 1-hour in-person closed beta-testing session, and 88% (23/26) of participants completed the anonymous questionnaire and were included in this study. Regarding participant satisfaction, Virtual Doc was enjoyed by 91% (21/23) of participants, with 74% (17/23) intending to recommend the simulation to a colleague and 66% (15/23) intending to recommend the simulation to a friend. In assessment of the perceived educational value of Virtual Doc, 70% (16/23) of participants agreed they had an improved understanding of cardiopulmonary resuscitation, and 78% (18/23) agreed that Virtual Doc will help prepare for and deal with real-life clinical scenarios. Furthermore, 91% (21/23) of participants agreed with the development of additional Virtual Doc cases as beneficial for learning. An evaluation of the game play features as our secondary objective revealed that 70% (16/23) of participants agreed with ease in understanding how to use Virtual Doc, and 74% (17/23) found the game play elements useful in understanding cardiopulmonary resuscitation. One-third (7/23, 30%) found it easy to work with the interactive elements. In addition, 74% (17/23) were interested in interacting with other students within the simulation.

**Conclusions:**

Our study demonstrates a positive response regarding trainee satisfaction and perceived educational efficacy of Virtual Doc. The simulation was widely accepted by the majority of users and may have the potential to improve educational learning objectives.

## Introduction

Simulation-based education plays a significant role in developing vital clinical assessment and management skills, especially in emergency scenarios such as a sudden cardiac arrest (SCA). SCA is an uncommon but life-threatening condition within the pediatric population [1,2] that includes the potential catastrophic consequences of mortality and poor neurological morbidity. An estimated 16,000 [3,4] children in the United States experience out-of-hospital SCAs and 5800 [4] experience in-hospital SCAs every year. Overall, survival rates are better for in-hospital arrests compared with out-of-hospital arrests [5], which is unsurprising considering that the most significant predictor of survival is early initiation of adequate cardiopulmonary resuscitation (CPR) [6]. However, junior doctors often have limited real-world learning experience managing an acutely ill and vulnerable child [7], especially in emergent scenarios, where active participation is often reserved for more experienced practitioners. In addition, many pediatric health care professionals feel inadequately prepared to manage a critically ill child, increasing the risk of medical errors [8]. Therefore, simulation training forms the cornerstone of resuscitation education, providing an opportunity for trainees to prepare for real-world clinical practice without risking patient safety [9-11]. However, in-person simulation training sessions are expensive and resource-intensive [12], and, therefore, the efficacy of alternative modalities should be explored.

The revolutionary development of highly immersive digitized learning resources such as virtual reality simulators harness the advantages of simulation while offering a cost-effective and widely accessible educational platform. Several studies have provided evidence for the use of virtual reality simulators for procedural skills training in the surgical field, with improved intraoperative performance [13] and accuracy [14] and a reduction in operating times [13] and errors [15-18]. One study investigated the use of virtual reality for CPR education compared with traditional mannequins and found virtual reality could be a valid and acceptable training method [19]. Furthermore, in a study by Wong et al [20], clinical CPR instructors outlined the limits of traditional education and noted the potential beneficial features of virtual reality as an additional learning tool. The experiential nature of virtual reality may also be conducive to developing procedural skills such as CPR, as repetitive hands-on practice permits the development of muscle memory [21], leading to technical skill competence. CPR involves the activation of declarative memory to recall the sequential steps and procedural memory to perform the active steps such as chest compressions [22], both of which may be developed and reinforced through the use of virtual reality.

To the best of our knowledge, there are no highly immersive virtual reality simulators for pediatric CPR training. As such, we developed Virtual Doc, a highly immersive and experiential 3D multimedia sensory virtual environment designed to teach pediatric CPR skills to medical students and junior doctors. We performed a prospective closed beta-testing study on undergraduate medical students with the primary aim to evaluate participant satisfaction and perceived educational efficacy of Virtual Doc. The secondary aim of this study was to assess the gameplay features of our simulation. We hypothesized that Virtual Doc would be an enjoyable and highly educational learning experience. We also hypothesized that the gameplay features would be beneficial in the facilitation of a realistic hands-on clinical learning experience.

## Methods

### Study Design

We conducted a prospective, uncontrolled, interventional closed beta-testing study in 2018 to assess the usability, acceptability, and perceived educational effectiveness of Virtual Doc as a method for teaching pediatric CPR to medical students. All participants provided implied consent by expressing interest in the study, attending a 1-hour closed beta-testing session, and completing an anonymous postsession questionnaire (Multimedia Appendix 1). The study was approved by the University of New South Wales Human Research Ethics Committee (HC180484).

### Participants

Participants were eligible to partake in this study if they were currently enrolled in the 6-year undergraduate medical program at the University of New South Wales, Sydney, Australia. All medical students from the 6-year program were eligible and invited to participate in this study through an information email sent out by the University of New South Wales Medical Society administration. Students were instructed to contact the study investigators with their expression of interest. They were subsequently allocated to a 1-hour session in which they would engage with Virtual Doc and complete the postsession questionnaire. Participants were excluded from the analysis if they did not complete the questionnaire.

### Virtual Doc

**Figure 1:**
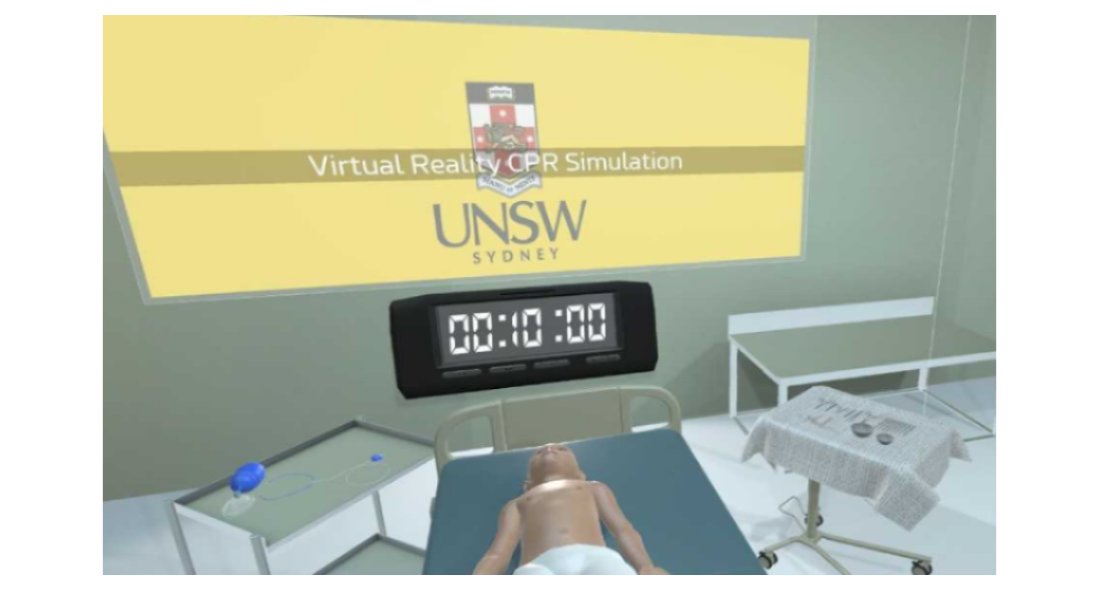
The Virtual Doc environment.

Virtual Doc is a virtual reality simulation developed for Oculus VR (Facebook Technologies LLC; Figure 1). The Virtual Doc app was developed using Unity3D software (Unity Technologies), and models were developed using open-source Blender modeling software. The simulation was conducted using the hardware of the Oculus Rift system linked to Alienware laptops (Alienware Corp). Virtual Doc is a first-person active learning experience through immersion within a multimedia sensory environment. Users wear a headset and use 2 hand controllers and are able to use these controllers, which are equipped with haptic technologies, to interact with the virtual environment. This simulation enables users to perform a series of actions including picking up objects, pushing buttons, and turning dials. They are also fully immersed with audiovisual aids such as auscultation of the heart and lungs.

**Figure 2:**
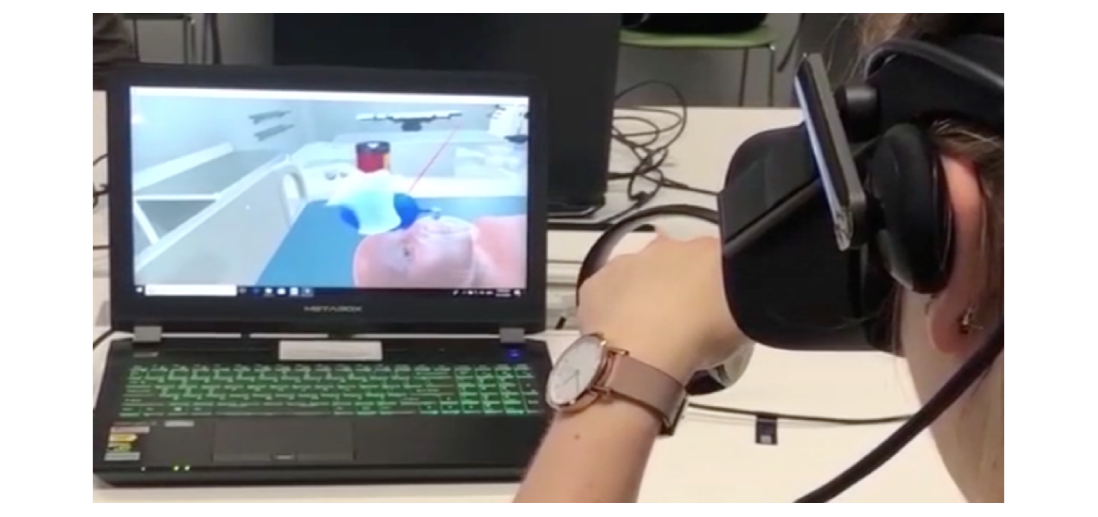
User is ventilating the patient with a bag-valve mask as per the resuscitation algorithm.

The scenario in this study involved an SCA in a young child that required appropriate and timely management. The required cardiac arrest management was in keeping with the Advanced Pediatric Life Support Australia guidelines [23]. Students were provided with a brief written clinical history on the main screen in the virtual environment and were then instructed to begin CPR. To complete the case, participants were required to assess the surroundings, check for a response, signal for senior help by pressing a call button, and then begin resuscitation by assessing the airway, breathing, and circulation; bagging and masking the patient (Figure 2); performing adequate chest compressions; and defibrillating the patient. As this scenario featured a shockable rhythm, this patient was defibrillated as part of the SCA management algorithm; however, this step may not be required for all pediatric SCA presentations. Shortly after the participant signaled for help, a senior physician arrived in the virtual environment to resume chest compressions while the user prepared for defibrillation. For successful completion of the case, all steps must have been completed correctly within 10 minutes.

### Outcome Measures

Validating the use of highly immersive and experiential virtual reality simulators as a form of active learning can be achieved by assessing various outcomes through the Kirkpatrick model of evaluation [24]. This 4-tiered hierarchy includes a scaffolding evaluation of reaction (level 1), learning (level 2), behavior (level 3), and results (level 4) [24]. As the levels progress, there is an increase in conclusively defining the efficacy of the educational activity [25].

The primary outcomes of this study were participant satisfaction (Kirkpatrick level 1) and the perceived educational value of Virtual Doc (Kirkpatrick level 2). Participant satisfaction was assessed through 1 Likert-style question and 2 trichotomous questions regarding enjoyment of the game and whether the participant would recommend the game to a colleague or friend. The perceived educational efficacy was assessed through 3 Likert-style questions including whether participants improved their understanding of the simulation objectives, whether engaging with Virtual Doc prepares students for a similar real-life clinical scenario, and whether different clinical cases would be beneficial for learning.

The secondary outcome was an evaluation of the gameplay features. Here, subjects were asked to respond to 3 Likert-style questions and 1 dichotomous question regarding the ease of understanding how to engage with the simulation, the level of ease to work with the interactive elements, whether the interactive elements were useful for learning, and whether the user would like to interact with other students in the virtual world.

### Testing Session

All interested participants were allocated to a 1-hour in-person session. Attendees were invited to attempt at least one full clinical scenario using the virtual reality simulator. They were offered an unlimited number of attempts within the 1-hour session. After engaging with Virtual Doc, participants were invited to complete an anonymous questionnaire that evaluated the primary and secondary outcomes.

### Data Collection and Statistical Analysis

Each postsession questionnaire was assigned a unique identification number and was completed anonymously by participants. The responses were collated and outcome measures were analyzed using descriptive statistics. The 7-point Likert scale responses were streamlined to 3 categories of agree (included all responses indicating strongly agree, agree, and mildly agree), neutral, and disagree (included all responses indicating mildly disagree, disagree, and strongly disagree) for the data analysis. Trichotomous questions requiring a response of yes, not sure, or no and dichotomous questions requiring a response of yes or no were analyzed as such. The data were analyzed using the statistical software R (version 1.1.423, R Foundation for Statistical Computing) [26].

## Results

A total of 26 participants were recruited and attended 1-hour in-person closed beta-testing sessions. A total of 88% (23/26) of participants completed the anonymous questionnaire and were included in the analysis, and 12% (3/26) attended the testing session but declined to complete the questionnaire and were therefore excluded from the analysis. They were not required to provide a reason for declining to participate.

### Study Population

All 23 of the included participants completed the demographic characteristics section of the questionnaire. Overall, the median age of participants was 22.0 (interquartile range 21.5-23.0) years, and 70% (16/23) of participants were female. In 2018, 61% (14/23) of participants were in year 4, 13% (3/23) were in year 5, and 26% (6/23) were in year 6. A total of 87% (20/23) of participants were local students, while 13% (3/23) were international students.

### Outcome Measures

**Figure 3:**
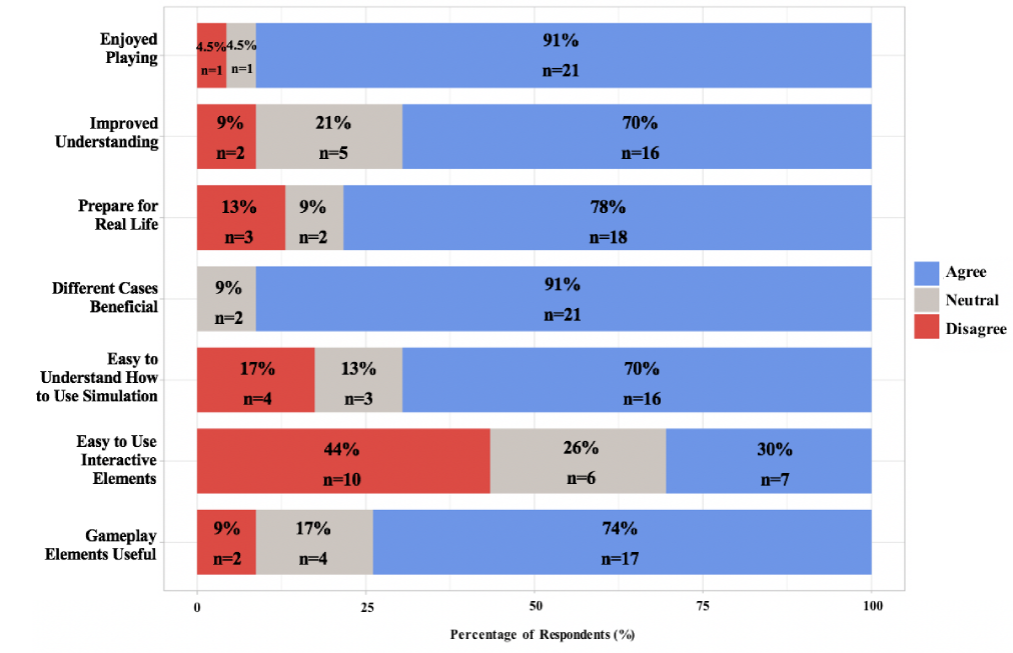
Results of the Likert-style questions.

**Figure 4:**
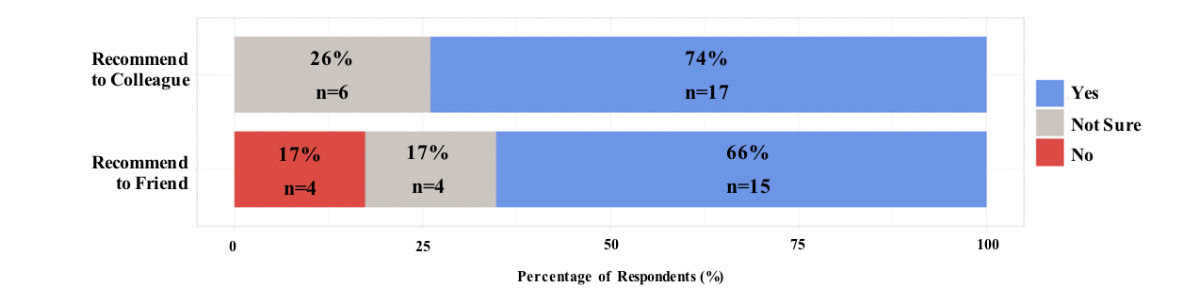
Results of the trichotomous questions.

**Figure 5:**
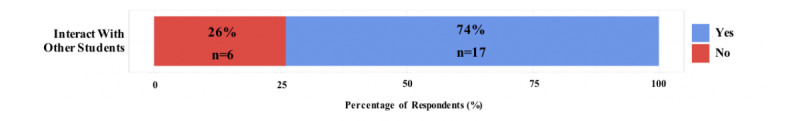
Results of the dichotomous question.

Participants responded to 10 Likert-style, trichotomous, or dichotomous questions regarding their experience with Virtual Doc. The results of these questions are summarized by question type in the horizontal stacked bar graphs in Figures 3-5.

### Participant Satisfaction

Respondents completed 1 Likert-style question and 2 trichotomous questions to evaluate satisfaction with Virtual Doc as an assessment of Kirkpatrick level 1. Virtual Doc was enjoyed by 91% (21/23) of participants. Furthermore, 74% (17/23) would recommend this simulation to a colleague, and 66% (15/23) would recommend this simulation to a friend.

### Perceived Educational Value

Participants completed 3 Likert-style questions to assess the perceived educational efficacy of Virtual Doc, evaluating Kirkpatrick level 2, and 70% (16/23) of participants agreed they had an improved understanding of CPR following the use of Virtual Doc. In addition, 78% (18/23) agreed that Virtual Doc will help them prepare for and deal with real-life clinical scenarios. Furthermore, 91% (21/23) agreed there was a learning benefit to developing different cases within Virtual Doc, and 9% (2/23) were neutral.

### Gameplay Features

Respondents completed 3 Likert-style questions and 1 dichotomous question evaluating the gameplay features of Virtual Doc. In terms of usability and gameplay, 70% (16/23) of participants agreed they had ease in understanding how to use Virtual Doc, and 74% (17/23) found the gameplay elements useful for understanding CPR. Furthermore, 30% (7/23) of participants found it easy to work with the interactive elements, which provides a template for future software improvements. In addition, 74% (17/23) would like the option to interact with other students within the game.

## Discussion

### Principal Findings

To the best of our knowledge, a prospective study on the use of a virtual reality simulator to teach pediatric CPR to medical students has yet to be conducted. In accordance with the Kirkpatrick model [24], the results of our study demonstrated high trainee satisfaction (Kirkpatrick level 1) and a perceived improvement in learning (Kirkpatrick level 2). In evaluating our secondary outcome of assessing the response to the virtual reality gameplay features, the majority of participants agreed with the ease in understanding how to use the simulation and the usefulness of gameplay features in improving their understanding of CPR and expressed a desire for interacting with others in the virtual environment. Although some participants found it difficult to work with the interactive elements, this closed beta test provided an opportunity to identify elements for future improvements, including the refinement of the interactive elements and future considerations such as a multiplayer design which may further enhance the learning experience and element of realism given the significant role of teamwork in resuscitation. Current data on trainee satisfaction, educational efficacy, and gameplay elements of virtual reality simulators for pediatric medical education is limited, and the encouraging results of this study will address the gap in the literature and could have an integral role in transforming the culture of future medical education.

Immersive technologies provide users with an engaging and enjoyable learning platform to train skills to proficiency without risking patient safety. Our study illustrated high participant satisfaction as Virtual Doc was enjoyed by almost all participants. This result parallels a study by Kron et al [27] in which an evaluation of medical student attitudes toward technology-based education was conducted and revealed that almost all students liked the idea of using technology to enhance their educational experience and thought that education should make better use of new media technologies. Beyond satisfaction, a previous study reported that experiential simulations offer a high level of interactivity and engagement, which may increase user motivation to learn [28]. Providing a highly engaging environment, such as that of Virtual Doc, is important for trainees as increased interest and satisfaction with the learning modality may result in an increased level of motivation to learn; one study showed that highly motivated students are more effective learners [29]. The importance of satisfaction and effective learning is reinforced by a study evaluating the effectiveness of virtual reality versus traditional or other forms of digital learning in educating health care professionals, in which a higher level of interactivity was more effective for the development of postintervention knowledge and skills [30]. The level of satisfaction with a learning experience also affects the probability of a user recommending such an experience to their peers [31]. The results of our study are congruent with this notion, as indicated by most students enjoying the simulation and agreeing with the intention of recommendation.

In this study, Virtual Doc was perceived as educationally efficacious by the majority of the participants, as indicated by a positive response toward improved understanding, preparation for real-world clinical scenarios, and support for designing additional cases. An important component of effective education in the adult learner involves the psychological concept of flow. This term is defined as a state in which a learner is “fully engaged, focused, and committed to the success of the activity” [32], and this can be achieved through active engagement in an experiential learning process. The use of highly immersive and interactive virtual reality simulators can improve flow, which may lead to superior learning outcomes and performance as echoed by the majority of participants reporting an improved understanding of CPR. Additionally, engaging sensory modalities including vision, haptics, and audition also promotes active learning, which can improve memory retention [33]. Virtual Doc includes sensory features through auscultation of heart and lung sounds and palpation of the peripheral pulses, which the user will hopefully be able to translate into the real-world clinical setting when assessing or managing a similar patient presentation.

Being immersed in the virtual yet realistic hospital environment of Virtual Doc provides users with a lifelike clinical experience. This digital-based environment is an innovative tool with realistic features supporting the recommendations of adult learning theories such as Knowles’ theory [34,35]. The responses in this study regarding the Virtual Doc environment and its ability to prepare students for real-life clinical scenarios showcases a facility in which the elements of adult learning theories can be included. Active experimentation within a realistic and immersive learning environment allows users to make clinical decisions and experience the consequences of their actions in real time, mimicking a real-world clinical environment. This element of realism is important in encouraging intrinsic motivation to support the needs of an adult learner.

Additionally, the Kolb cycle of learning encourages repetition, reflection, and correction to improve learning outcomes [36]. Virtual Doc supports this cyclical relationship as learners have the opportunity to engage in repetitive practice until they achieve educational mastery in a safe and controlled environment, which is an important benefit of simulation as this is severely limited in real-world emergency patient encounters, especially in the pediatric population [5]. The flexible design of virtual reality may enable health care professionals to have more convenient and frequent opportunities to practice and refine skills.

Several studies have demonstrated that junior doctors feel inadequately prepared and lack confidence in their early training years [37-41]. This is further supplemented by less favorable outcomes for neonatal resuscitation being described in the summer when senior staff physicians are less likely to be present and the overall volume of staff members is reduced [42]. Lacking confidence and inadequate preparation may translate into unsafe practice and poor patient outcomes and is an especially important consideration when managing emergency scenarios such as an SCA. Virtual reality simulators such as Virtual Doc may provide an avenue to bridge the gap between trainee and doctor through immersive gameplay by enabling users to master foundational principles and basic management algorithms and apply this knowledge to challenging simulated cases. In our study, the majority of respondents expressed that this simulation game would aid in developing clinical skills and preparing the user for dealing with real-life clinical scenarios. In addition, almost all participants expressed benefit in the development of different cases, indicating a positive response toward the clinical educational value of the simulation.

The gameplay elements involved in virtual reality add entertainment to the learning process, thereby motivating learners to engage in study. The triad of immersion, interaction, and imagination, as described by Burdea and Coiffet [43], are important factors of virtual reality technology. Coupled with andragogical principles, a highly interactive game design improves intrinsic motivation and creates a more enjoyable and engaging learning experience with a consequential potential to illustrate superior examination scores [44]. In our study, the vast majority of respondents agreed that the interactive gameplay elements were useful in understanding how to perform CPR and found the process of the virtual reality simulation easy to understand. This was further supplemented by almost all participants enjoying the experience in the assessment of participant satisfaction. However, some respondents did not experience ease in using the interactive elements within the virtual environment. As this was a closed beta test, the software was able to deliver its intentional value to the participants, but there were some minor issues reported with ease in fully integrating into the environment such as accurately picking up a stethoscope or pushing the assistance button. This feedback is being considered as we progress toward the final product and acts as identified areas for future developments.

Finally, nontechnical skills such as teamwork and leadership are paramount in successful resuscitation. The collegiality of the responding team directly impacts predictors of postarrest survival as a coordinated course of action is associated with improved patient outcomes [45]. Technology-based games such as Virtual Doc have the potential to facilitate an atmosphere of teamwork, and in the digital era, medical students have agreed with introducing multiplayer simulations if they were fun and developed clinical skills [27]. Likewise, our study demonstrated that the majority of participants were interested in interacting with other players. A multiplayer design within the virtual environment could afford an opportunity to rotate through different resuscitation team roles, supporting an active and immersive learning experience, with the potential to equip learners with the crucial teamwork skills required for the effective management of a pediatric SCA.

### Limitations

Our study was limited by the small sample size and monocentric design, which limits its generalizability and external validity. However, participants from all years of study were eligible to participate, providing a degree of generalizability. This trial is also limited by voluntary convenience sampling, leading to a potential selection bias. Furthermore, this study used a single intervention with no active comparator, and therefore, the participants were not randomized and were unblinded to the intervention, which may formulate a measurement bias. However, the questionnaires were anonymized, providing a layer of security for participants to provide uncoerced feedback. Moreover, the final version of Virtual Doc will differ from the prototype used in this study as this was closed beta-testing and we are improving the software to address the feedback provided by the participants in this study. In addition, the prototype design of Virtual Doc in this closed beta test did not assess user hand technique or the quality of chest compressions. This limitation will guide future software improvements to ensure users are equipped with real-time visual and haptic feedback regarding the technique and quality of chest compressions.

### Future Research

Given the encouraging results of this study, future research should include an investigator-blinded randomized controlled trial to objectively evaluate the educational efficacy of Virtual Doc against traditional simulation-based education or other digitized modalities such as serious games or e-learning modules. Furthermore, an evaluation of translation of knowledge into clinical practice (Kirkpatrick level 3) and the impact of learned knowledge on changes within the organizational practice to improve patient outcomes (Kirkpatrick level 4) should be performed, with emphasis on a reduction in medical errors and improved patient survival rates and overall health outcomes. This study does not assess the higher levels of the Kirkpatrick model, and this limitation should be the focus of future research to contribute to the quality of data for the efficacy of Virtual Doc.

### Conclusion

In summary, our study demonstrates a positive response regarding the satisfaction and educational efficacy of Virtual Doc. Our findings reveal that this virtual reality simulation was widely accepted by the majority of users and has the potential to improve educational learning objectives. As such, our results provide a promising contribution to the educational revolution and may encourage the use of this emerging and versatile technology in the transformation of the 21st century medical curricula.

## Appendix

Multimedia Appendix 1 Questionnaire instrument.

## Abbreviations

CPR: cardiopulmonary resuscitation
SCA: sudden cardiac arrest
