# Supplementary material for: Resuscitating Cardiopulmonary Resuscitation Training in a Virtual Reality: Prospective Interventional Study
Source: J Med Internet Res. 2021 Jul 29;23(7):e22920. doi: 10.2196/22920 (PMC8367149; doi:10.2196/22920)
Supplement: Multimedia Appendix 1 [file jmir_v23i7e22920_app1.doc]

**Questionnaire – Virtual Doc VR Game**

Please answer the following questionnaire.

Please state your age: ________

Please circle your gender: Male / Female

Please circle your student type: Local / International

Please tell us in which year you did enrol in this degree: ___________

It was easy to understand how to play the video-game (tick one):

- Strongly agree
- Agree
- Mildly Agree
- Neutral
- Mildly Disagree
- Disagree
- Strongly disagree

The video-game helped improve my understanding of the topic (tick one):

- Strongly agree
- Agree
- Mildly Agree
- Neutral
- Mildly Disagree
- Disagree
- Strongly disagree

The video-game is going to help me prepare for and deal with real-life clinical scenarios (tick one):

- Strongly agree
- Agree
- Mildly Agree
- Neutral
- Mildly Disagree
- Disagree
- Strongly disagree

I found it easy to work with the interactive elements of the video-game (tick one):

- Strongly agree
- Agree
- Mildly Agree
- Neutral
- Mildly Disagree
- Disagree
- Strongly disagree

I believe different case based video-game scenarios (i.e. different cases for different medical presentations and diagnosis) will help with my learning (tick one):

- Strongly agree
- Agree
- Mildly Agree
- Neutral
- Mildly Disagree
- Disagree
- Strongly disagree

I found the gameplay elements (ability to interact with the game) useful in my understanding of the topic (tick one):

- Strongly agree
- Agree
- Mildly Agree
- Neutral
- Mildly Disagree
- Disagree
- Strongly disagree

Would you like to interact with other students in the video-game (tick one)?

- Yes
- No

I enjoyed playing the video-game:

- Strongly agree
- Agree
- Mildly Agree
- Neutral
- Mildly Disagree
- Disagree
- Strongly disagree

Would you suggest this video-game to a colleague studying Paediatrics or Medicine (tick one)?

- Yes
- No
- Not sure

In general, would you suggest this video-game to a non-medical student friend (tick one)?

- Yes
- No
- Not sure
